# Assessment of Pathogen Contamination in Food at Teaching Hospitals in Mashhad, Iran: A Cross‐Sectional Study

**DOI:** 10.1002/hsr2.72962

**Published:** 2026-08-26

**Authors:** Mojtaba Raeisi, Fateme Asadi Touranlou, Golshan Shakeri, Maliheh Doustinouri, Arefeh Erfani, Asma Afshari, Mohammad Hashemi

**Affiliations:** ^1^ Food, Drug and Natural Products Health Research Center Golestan University of Medical Sciences Gorgan Iran; ^2^ Students Research Committee Tehran University of Medical Sciences Tehran Iran; ^3^ Division of Food Safety and Hygiene, Department of Environmental Health Engineering, School of Public Health Tehran University of Medical Sciences Tehran Iran; ^4^ Medical Toxicology Research Center Mashhad University of Medical Sciences Mashhad Iran; ^5^ Department of Nutrition, Faculty of Medicine Mashhad University of Medical Sciences Mashhad Iran

**Keywords:** bacterial contamination, food pathogens, hospital food, polymerase chain reaction

## Abstract

**Background and Aims:**

Infectious diseases in hospitals pose a significant threat, with some linked to contaminated food. Food safety is a critical issue for hospital patients, particularly those with weakened immune systems. This study aimed to conduct the first comprehensive microbial evaluation of hospital catering across 12 teaching hospitals in Mashhad, Iran.

**Methods:**

A total of 360 food samples from 10 distinct food categories (simple rice, mixed rice with meat or vegetables, barbecue chicken, kebab kubideh, chicken, fish, soup, stew, and raw vegetables) were analyzed. Integrated streaking culture and PCR techniques targeting the 16 s *InvA*, *prfA*, *eaeA*, *groEL*, and 16S rDNA genes were employed to confirm five key pathogens.

**Results:**

The prevalence of pathogens was as follows: *Salmonella* spp. (8.3%), *Listeria monocytogenes* (5.8%), *Escherichia coli* O157:H7 (1.1%), *Bacillus cereus* (0.6%), and *Clostridium perfringens* (0.6%). Raw vegetables exhibited the highest contamination (5.70 ± 0.46 log CFU/g, with 6 samples exceeding Iranian National Standards), followed by grilled foods, indicating cross‐contamination risks. Overall, 16.4% of samples (59/360) failed microbial quality standards, highlighting significant safety concerns for vulnerable immunocompromised patients.

**Conclusion:**

These findings highlight the urgent need for enhanced washing protocols for raw vegetables, adequate cooking temperatures, and staff training to ensure safe hospital catering.

## 1 Introduction

Foodborne diseasesMojtaba Raeisi remain a critical concern for public health, the economy, and society globally [1]. Despite various interventions by governmental and industrial sectors, the prevalence of foodborne illnesses remains alarmingly high in both developed and developing countries [2]. The World Health Organization (WHO) reported in 2015 that approximately 1 in 10 individuals globally fall ill each year due to contaminated food, resulting in 600 million cases of illness, 420,000 deaths, and a loss of 33 million healthy years of life [3, 4]. In Iran, food safety is a pressing public health issue, with the incidence of foodborne diseases recorded at 1.38 cases per 100,000 individuals as of 2011 [5, 6], reflecting a serious public health challenge that persists today.

Hospitals play a pivotal role in ensuring patient safety and must adhere to stringent guidelines to maintain a hygienic environment [7]. The microbial quality of food served within healthcare facilities is particularly important, as vulnerable populations, such as immunocompromised patients, face heightened risks for foodborne illnesses [8]. Understanding the microbial quality of hospital food is essential to prevent these illnesses, especially among patients with compromised immune systems [9]. Factors contributing to food contamination in hospital settings include improper storage of raw materials, insufficient cooking, inadequate cooling practices, extended food storage, and cross‐contamination due to poor hygiene among food handlers [10]. Therefore, adherence to standards and protocols like Good Manufacturing Practices (GMP), Good Hygiene Practices (GHP), and Hazard Analysis Critical Control Points (HACCP) is crucial in mitigating the risk of infections associated with contaminated food [11, 12].

Research indicates that common pathogens responsible for foodborne illnesses, such as *Staphylococcus aureus*, *Salmonella*, *Clostridium perfringens*, *Listeria monocytogenes*, *Campylobacter*, *Escherichia coli*, *Bacillus cereus*, *Shigella*, and *Vibrio parahemolyticus*, are often found in raw or undercooked foods [13, 14]. These bacterial infections significantly impact public health, leading to symptoms such as diarrhea, fever, headache, vomiting, abdominal cramps, and extreme fatigue [15]. In Iran, studies have highlighted the prevalence of these pathogens in healthcare facilities. For example, a study in a Tehran teaching hospital revealed that 15.5% of kitchen samples were contaminated with *S. aureus*, while other pathogens such as *E. coli* and *Acinetobacter* spp. were also detected [16]. Additionally, research in Mashhad indicated that an alarming 91.6% of blenderized tube feeding (BTF) samples were contaminated with bacteria, including *E. coli*, *Salmonella* spp., and *L. monocytogenes* [17]. These findings underscore the significant challenges in maintaining food safety within Iranian hospitals, particularly concerning the handling of medical foods.

Given that approximately 90% of patients depend on hospital catering for their nutritional needs, it is imperative that hospital caterers adhere to established food safety standards. However, pervasive issues such as outdated equipment and economic constraints often hinder the ability to provide safe and nutritious meals [18]. Despite the critical importance of microbial evaluation in hospital catering services, there is a notable scarcity of such studies in Iran, particularly in regions like Mashhad. This gap highlights the urgent need for further research to assess the microbial quality of cooked meals and raw vegetables served to patients in teaching hospitals, thereby ensuring the safety and health of this vulnerable population.

## 2 Materials and Methods

### 2.1 Sampling

A total of 360 food samples were collected randomly from 10 common menu items provided by the catering services of teaching hospitals (H1‐H12) in Mashhad, in three replications. The samples included barbecue chicken, kebab kubideh (made from ground beef or lamb), simple rice, rice mixed with meat, rice mixed with vegetables, soup, poultry, fish, stew, and raw vegetables. The collected samples were immediately transferred to the laboratory of the Nutrition Department at Mashhad Medical School under aseptic conditions and stored under cold conditions until analysis.

### 2.2 Microbial Analyses

#### 2.2.1 Isolation and Identification of Salmonella

Following the guidelines of the Iranian National Standard Organisation no. 2394, 25 g of each sample was added to 225 mL of sterile Buffered Peptone Water (BPW) (Merck, Darmstadt, Germany) and incubated at 37°C for 18–24 h for pre‐enrichment. Subsequently, 1 mL of the incubated BPW was added to 9 mL of selenite cysteine (SC) broth (Liofilchem, Roseto Degli Abruzzi, Italy) and incubated at 37°C for 24 h. A loopful from each enriched broth was then streaked onto Xylose Lysine Desoxycholate (XLD) agar (Merck) and incubated at 37°C for 18 to 24 h [19]. Typical suspected *Salmonella* colonies on XLD agar appear red with or without black centers due to hydrogen sulfide production. Suspected colonies were selected and subjected to confirmation testing by culturing them in Triple Sugar Iron Agar (Merck) and incubating at 37°C for 18–24 h. Colonies with black centers and colorless peripheries were identified as Salmonella and confirmed by PCR assay [17] (Table 1).

**Table 1:** Sequence of oligonucleotides used as primers in the PCR.

Species of bacteria	Sequences (5′–3′)	Target gene	Product size (bp)	References
*L. monocytogenes*	F: TCA TCG ACG GCA ACC TCG G	*prfA gene*	217	[20]
	R: TGA GCA ACG TAT CCT CCA GAG T			
*Salmonella* spp.	F: GTGAAATTATCGCCACGTTCGGGCAA	*InvA*	284	[17]
	R:TCATCGCACCGTCAAAGGAACC			
*C. perfringens*	F: AAAGATGGCATCATCATTCAAC	*16S rDNA*	279	[21]
	R: TACCGTCATTATCTTCCCCAAA			
*B. cereus*	F: GTG CGA ACC CAA TGG GTCTTC	*groEL gene*	400	[22]
	R: CCT TGT TGT ACC ACT TGC TC			
*E. coli* O157 H7	F: GGC GGA TAA GAC TTC GGCTA	*EaeA gene*	151	[21]
	R: CGT TTT GGC ACT ATT TGC CC			

#### 2.2.2 Identification of Listeria Monocytogenes

The identification and isolation of *L. monocytogenes* were performed following the ISO 11290 method modified by Jamali et al. [23]. Initially, 25 g of samples were combined with 225 mL of half Fraser broth (Oxoid, Basingstoke, UK) in a stomacher bag for the first enrichment step. The mixture was homogenized using a stomacher (Lab blender 400, Seward Medical, London, UK) and incubated at 30 ± 1°C for 24 h. A loopful of the initial enriched broth culture was streaked on CHROMagar *Listeria* (CHROMagar, Paris, France) and further incubated at 37°C for 24‐48 h. In addition, 0.1 mL of half Fraser broth (Oxoid) was introduced into 10 mL of half Fraser broth (Oxoid) for the second enrichment phase, incubated at 37°C for 48 h. A loopful of the enriched Fraser broth culture was then streaked onto PALCAM agar (Oxoid) and incubated for another 24‐48 h at 37°C. *Listeria* colonies on PALCAM agar typically appear grayish‐green with a characteristic black halo due to lecithinase production. Identification of colonies was confirmed through PCR targeting the *PrfA* gene (Table 1).

#### 2.2.3 Escherichia Coli O157 H7

The analysis for the presence of *E. coli* O157 H7 in the samples was conducted following the FDA Bacteriological Analytical Manual (BAM) [24]. Twenty‐five grams of each sample were aseptically introduced into 225 mL of Brain Heart Infusion (BHI) broth (Quelab, Montreal, Canada) and homogenized in a stomacher for 1 min. The resulting suspension was then incubated at 35°C for 3 h. Following this, 225 mL of double‐strength tryptone phosphate (TP) broth (Quelab) was added, and the mixture was further incubated at 44°C for 20 h as part of the enrichment process. A single loopful from each stomacher bag was streaked onto MacConkey agar (Merck) and then onto Levin‐Eosin Methylene Blue (L‐EMB) agar (Quelab) [25]. On MacConkey agar, *E. coli* O157 H7 colonies appear as colorless or pale colonies due to the inability to ferment lactose. On l‐EMB agar, the colonies typically exhibit a dark (metallic sheen) appearance due to lactose fermentation. Colony identification was verified through PCR targeting the *EaeA* gene (Table 1).

#### 2.2.4 Enumeration and Identification of *Bacillus cereus*

*B. cereus* was detected using the Iranian Standard Method no. 2324 [26]. Samples were serially diluted (10^−1^ to 10^−6^) in sterile 0.1% peptone water. One milliliter of each dilution was spread in duplicate onto Mannitol‐egg yolk‐polymyxin (MYP) agar and incubated at 30°C for 48 h. This agar medium contains an egg yolk emulsion and polymyxin B sulfate (Shijiazhuang Pharma, Shijiazhuang, China). Large pink colonies, indicating a lack of mannitol fermentation, with a surrounding halo, indicating lecithinase production, were considered potential *B. cereus* colonies. To confirm the identity of these suspected colonies, a hemolysis test was conducted on blood agar (Merck). Colony identification was validated through PCR by detecting the *B. cereus GroEL* gene, with results reported in CFU/g [27].

#### 2.2.5 Clostridium perfringens

During this phase, 10 g of each sample was thoroughly mixed with 90 mL of BPW (Merck), and then 1 mL was transferred to a tube containing 9 mL of fluid thioglycollate (FTG, HiMedia; Merck) [5]. The tubes were placed in anaerobic conditions and incubated at 32°C for 15–24 h. After initial enrichment of *C. perfringens*, each tube was streaked onto blood agar medium (Merck), which typically exhibits double‐zone hemolysis, and incubated at 45°C for 32 h in an anaerobic jar. To confirm the presence of *C. perfringens*, a loopful was spread onto tryptose sulfite cycloserine agar (Sigma–Aldrich, Missouri, United States) from colonies exhibiting duplicate hemolysis on the blood agar medium. *C. perfringens* colonies on tryptose sulfite cycloserine agar are typically black due to sulfite reduction, and the plates were subsequently incubated at 32°C for 24 h under anaerobic conditions.

### 2.3 Extracting DNA

The boiling method was employed for DNA extraction [17]. Two typical, well‐isolated colonies (approximately 2–3 mm in diameter) from 18 to 24 h cultures on selective media were picked and placed in a tube containing 50 mL of distilled water and boiled in a water bath at 100°C for 10 min. Subsequently, the tubes were centrifuged for 5 min at 13,000 rpm, and 5 μL of the supernatant was transferred into a separate sterile tube (1.5 mL). A nanodrop spectrophotometer (model: 2000 UV‐Vis, Thermo Fisher Scientific, Deutschland, Germany) was used to measure the DNA concentration before proceeding with PCR.

### 2.4 PCR Amplification

Colonies observed on selective media and confirmed by biochemical tests served as templates for the PCR assay. The positive controls included *E. coli* O157 H7 (PTCC‐1338), *Salmonella* spp. (PTCC‐1609), *L. monocytogenes* (ATCC‐7644), *C. perfringens* (ATCC‐1312), and *B. cereus* (ATCC‐10876), while sterile distilled water was used as the negative control. Each microtube contained a final volume of 25 μL, comprising 12.5 μL of Taq 2× red master with MgCl2 (1.5 mM, Amplicon, Odense, Denmark), 1 μL of forward primer, 1 μL of reverse primer (refer to Tables 1), 5.5 μL of sterile distilled water, and 5 μl of DNA extracted from the target bacteria [17]. The DNA templates from suspected isolates were analyzed through six PCR steps: initial denaturation, denaturation cycles, annealing, extension, and a final extension cycle. The amplification reactions and cycling parameters were applied to the food samples using thermal cyclers (BIO‐Rad, Hercules, California) as detailed in Table 2. After these procedures, PCR products were loaded onto a 1.5% (w/v) agarose gel (Sigma–Aldrich). The gel was stained with a green viewer (Parstous Biotechnology, Mashhad, Iran), and gel electrophoresis was performed at a constant voltage of 110 V for 40 min. Finally, the gels were visualized under UV light with a gel imaging system (Uvi tech, England, United Kingdom).

**Table 2:** The thermal program of the thermocycler.

Species of bacteria	Initial denaturation	Denaturation	Annealing	Extension	Final extension	Cycles	References
*Salmonella* spp.	95°C 5 min	94°C 1 min	56°C 30 s	72°C 30 s	72°C 10 min	35	[28]
*Listeria monocytogenes*	95°C 7 min	95°C 50 s	54°C 40 s	72°C 50 s	72°C 5 min	40	[17]
*E. coli* O157 H7	95°C 5 min	95°C 30 s	60.5°C 1 min	72°C 30 s	72°C 10 min	35	[25]
*Clostridium perfringens*	95°C 5 min	94°C 1 min	56°C 1 min	72°C 1 min	72°C 5 min	40	[17]
*Bacillus cereus*	95°C 5 min	94°C 30 s	63°C 30 s	72°C 30 s	72°C 5 min	30	[22]

### 2.5 Statistical Analysis

Descriptive statistics, including the mean and standard deviation (SD), were used to summarize the counts. Analyses were performed using SPSS version 22.0 (SPSS Inc., Chicago, IL, USA). To compare the mean Total Bacterial Counts (TBC) and *B. cereus* counts across the 12 participating hospitals and the 10 food categories, One‐way Analysis of Variance (ANOVA) was performed. Statistically significant differences were further explored using Tukey's Honest Significant Difference (HSD) test for multiple pairwise comparisons. A significance level of *p* < 0.05 was considered statistically significant [29].

### 2.6 Ethics Approval

The study protocol was approved by the Ethics Committee of Mashhad University of Medical Sciences (IR.MUMS.MEDICAL.REC.1397.420) after obtaining the required permit for the research.

## 3 Results and Discussion

Foodborne diseases pose a significant global health concern, particularly in hospitals and medical centers [30]. Since foodborne pathogens can worsen the clinical conditions of hospitalized patients, it is crucial for hospital kitchens to implement stringent food safety protocols to identify and manage food hazards [31]. Regular checks and monitoring of food served in hospitals are essential to prevent and minimize foodborne illnesses, especially among vulnerable populations [32].

In this study, we assessed microbial contamination levels in food samples collected from 12 teaching hospitals in Mashhad, comparing the findings to the Iranian National Standards (Table 3). Our analysis revealed that raw vegetable samples, particularly salads, had the highest contamination levels, averaging 5.7 ± 0.46 log CFU/g (Tables 4 and 5). In contrast, cooked foods, including koubideh kebab, chicken, fish, meat stews, rice, and soups, showed significantly lower levels of microbial contamination. Notably, only six raw vegetable samples from hospitals H1, H4, H6, H7, H10, and H11 exceeded the microbial limits, highlighting the urgent need to improve food handling and preparation practices in hospital kitchens. Statistical analysis indicated significant differences (*p* < 0.05) in total bacterial counts among raw vegetables, chicken, stews, and soups across the 11 hospitals, suggesting variability in contamination sources or handling practices.

**Table 3:** Microbial contamination limits of food samples according to Iranian National Standards (CFU/g).

Food sample	Microbial Contamination Limits (log CFU/g)				
	Total count	*Bacillus cereus*	*Escherichia coli O157 H7*	*Listeria monocytogenes*	*Salmonella*
Simple rice	5	1>	Negative	Negative	Negative
Rice mixed with vegetable	5	1>	Negative	Negative	Negative
Rice mixed with meat	5	1>	Negative	Negative	Negative
Chicken barbecue	5	—	Negative	Negative	Negative
Kebab barbecue	5	—	Negative	Negative	Negative
Chicken	4	—	Negative	Negative	Negative
Fish	4	—	Negative	Negative	Negative
Soup	5	—	Negative	Negative	Negative
Stew	5	—	Negative	Negative	Negative
Raw vegetable	6	—	Negative	Negative	Negative

**Table 4:** Total count of bacteria (mean ± SD) in food samples of catering hospitals in Mashhad (log CFU/g).

Hospital	N*	Simple rice	Rice mixed with vegetables	Rice mixed with meat	Kubideh kebab	Chicken barbecue
H1	30	2.34 ± 0.49	2.28 ± 0.48	2.88 ± 0.77	2.44 ± 0.42	**3.12** ± **0.82**
H2	30	2.35 ± 0.62	2.44 ± 0.53	2.10 ± 0.17	2.1 ± 0.17	**2.25** ± **0.24**
H3	30	2.89 ± 0.37	2.53 ± 0.80	3.16 ± 0.71	3.75 ± 0.93	**3.30** ± **0.53**
H4	30	2.1 ± 0.17	2.15 ± 0.80	2.25 ± 0.24	2.3 ± 0.3	**2.38** ± **0.42**
H5	30	3.1 ± 0.17	2.98 ± 0.25	2.44 ± 0.42	2.72 ± 0.38	**2.96** ± **0.17**
H6	30	2.3 ± 0.17	2.76 ± 0.32	2.25 ± 0.24	3.01 ± 0.43	**2.41** ± **0.1**
H7	30	2.66 ± 0.76	2.30 ± 0.32	2.56 ± 0.73	3.01 ± 0.51	**2.4** ± **0.34**
H8	30	2.25 ± 0.24	1.90 ± 0.16	2.23 ± 0.4	2.81 ± 0.71	**2.59** ± **0.65**
H9	30	2.1 ± 0.17	2.81 ± 0.31	2.12 ± 0.15	2.86 ± 0.83	**2.1** ± **0.17**
H10	30	2.3 ± 0.3	2.51 ± 0.48	2.1 ± 0.17	2.33 ± 0.35	**2.86** ± **0.67**
H11	30	2.69 ± 0.69	2.4 ± 0.17	2.28 ± 0.48	2.78 ± 0.69	**2.41** ± **0.39**
H12	30	2.2 ± 0.17	2.58 ± 0.27	2.33 ± 0.35	2.51 ± 0.89	**3.09** ± **0.19**
Total	360	2.43 ± 0.46	2.42 ± 0.36	2.39 ± 0.4	2.78 ± 0.58	**2.0** ± **63.39**
***p* value** **		**0.117**	**0.299**	**0.180**	**0.106**	0.057

* *N* = Number of samples for each hospital

** Significant difference (*p* < 0.05)

**Table 5:** Total count of bacteria (Mean ± SD) in food samples of catering hospitals in Mashhad (log CFU/g).

Hospital	Chicken	Soup	Stew	Fish	Raw vegetables (vegetable & salad)
H1	3.46 ± 0.66	3.04 ± 0.33	2.59 ± 0.54	2.4 ± 0.45	**6.92** ± **0.11** **
H2	2.61 ± 0.4	3.14 ± 0.55	2.85 ± 0.80	2.25 ± 0.24	**4.47** ± **0.3**
H3	2.56 ± 0.54	3.45 ± 0.57	2.33 ± 0.35	3.21 ± 0.85	**3.37** ± **0.1**
H4	2.34 ± 0.6	3.88 ± 0.53	2.44 ± 0.53	2.94 ± 0.94	**6.57** ± **0.68** **
H5	2.38 ± 0.42	2.38 ± 0.42	4.37 ± 0.67	2.25 ± 0.44	**5.1** ± **0.3**
H6	2.71 ± 0	3.18 ± 0.25	2.65 ± 0.89	—	**6.33** ± **0.23** **
H7	2. ± 0.17	2.54 ± 0.27	2.84 ± 0.75	2.46 ± 0.8	**6.19** ± **0.32** **
H8	3.1 ± 1.02	2.5 ± 0.98	2.25 ± 0.24	2.56 ± 0.51	**5.92** ± **0.88**
H9	2.35 ± 0.39	2.25 ± 0.24	2.1 ± 0.17	2.59 ± 0.37	**4.73** ± **0.64**
H10	3.58 ± 0.49	2.49 ± 0.09	2.25 ± 0.24	2.35 ± 0.31	**6.51** ± **0.42** **
H11	2.35 ± 0.31	2.59 ± 0.54	3.24 ± 0.66	2.71 ± 0.21	**6.94** ± **0.46** **
H12	2.72 ± 0.26	2.66 ± 0.49	2.43 ± 0.51	2.46 ± 0.8	**5.32** ± **0.13**
Total mean	2.67 ± 0.68	2.48 ± 0.68	2.37 ± 0.82	2.75 ± 0.56	**5.7** ± **0.46**
***p* value** *	**0.021**	**0.011**	**0.006**	**0.701**	0.001

* *p* < 0.05 is highly statistically significant; *p* < 0.001 is highly statistically significant.

** Samples exceeding Iranian National Standards (e.g., > 5.00 log CFU/g for raw vegetables, > 4.00 log CFU/g for cooked foods).

Several factors contribute to elevated bacterial counts in certain food types. Raw vegetables, often consumed without cooking, are particularly susceptible to environmental contamination from soil, water, or improper handling, and may not receive adequate washing or sanitization [33, 34]. Soups and stews, typically prepared in bulk and stored for extended periods, can encourage bacterial growth if not maintained at safe temperatures [35]. Chicken, being protein‐rich, is also vulnerable to contamination during slaughter, processing, or cooking when hygiene protocols are not strictly followed [36]. These findings corroborate previous research demonstrating that raw and minimally processed foods are common vectors for foodborne pathogens in healthcare settings [5, 37].

Of the 360 food samples analyzed (including rice, kebab, stew, soup, fish, chicken, and raw vegetables), 65 (18.2%) were contaminated with at least one of the assessed pathogens: *Salmonella*, *L. monocytogenes*, *C. perfringens*, *B. cereus*, and *E. coli* O157 H7 (Table 6). Raw vegetables exhibited the highest contamination frequency (33 cases), consistent with their exposure to environmental pathogens and limited processing. This aligns with reports from Iran indicating suboptimal microbial quality in ready‐to‐eat foods and salads [38]. Among cooked foods, grilled items like kubideh and chicken barbecue showed higher contamination rates compared to other menu options, likely due to cross‐contamination during the handling, storage, or preparation of raw meats prior to grilling [39].

**Table 6:** Frequency of evaluated bacteria in different food menus of catering hospitals in Mashhad.

Food samples		*Escherichia coli* O157 H7	*Bacillus cereus*	*Clostridium perfringens*	*Listeria monocytogenes*	*Salmonella*	Total count	Total (%)
Rice	Simple	0	0	0	0	2	0	2
	Mixed	0	0	0	1	2	0	3
Barbecue	chicken	1	0	0	3	3	0	7
	Kubideh	0	1	0	3	4	0	8
Chicken		0	1	0	2	2	0	5
Soup		0	0	0	1	2	0	3
Stew		0	0	0	1	1	0	2
Fish		0	0	1	3	2	0	6
Raw vegetables		3	0	1	7	12	6	33
Total		4 (1.1%)	2 (0.5%)	2 (0.5%)	21 (5.8%)	30 (8.4%)	6 (1.6%)	65 (18.2%)

The research by Ragab et al. [32] in Egypt found that 24.3% of processed hospital food samples, which included items such as rice, meat, chicken, and bread, and 42.0% of non‐processed hospital food samples, including cucumber, tomato, date, grape, and banana, demonstrated unsatisfactory levels of fecal coliform counts. Furthermore, *E. coli* was identified in 7.1% of processed samples and 30.6% of non‐processed samples, raising significant concerns regarding microbial quality. These results support our study's findings, which indicate a higher level of contamination in unprocessed foods.

The distribution of pathogens varied across hospitals (Table 7). *Salmonella* was most prevalent in hospitals H5, H1, H9, and H12, while *L. monocytogenes* was predominantly found in H8, H7, H5, H10, and H12. Hospital H2 had the lowest overall contamination, whereas H10 and H12 exhibited the highest levels. *Salmonella* and *L. monocytogenes* were identified as the most pathogenic, while *E. coli* O157 H7, *B. cereus*, and *C. perfringens* showed lower levels of contamination (Table 7).

**Table 7:** Frequency of bacteria in food samples of teaching hospitals in Mashhad.

Hospital	N*	*Escherichia coli* O157 H7	*Bacillus cereus*	*Clostridium perfringens*	*Listeria monocytogenes*	*Salmonella*	Total
H1	30	0	0	0	1	4	6 (20%)
H2	30	0	0	0	0	1	1 (3.3%)
H3	30	0	1	0	1	2	4 (13.3%)
H4	30	0	0	1	0	1	3 (10%)
H5	30	0	0	0	3	4	7 (23.3%)
H6	30	0	0	0	2	1	4 (13.3%)
H7	30	1	0	0	3	2	7 (23.3%)
H8	30	1	0	1	3	2	7 (23.3%)
H9	30	0	0	0	1	4	5 (16.6%)
H10	30	0	1	0	3	3	8 (26.6%)
H11	30	1	0	0	0	1	3 (10%)
H12	30	1	0	0	3	4	8 (26.6%)
Total	360	4	2	2	21	30	59 (16.4%)

* *N* = Number of samples for each hospital

The presence of pathogens like *Salmonella* emphasizes the need for stringent food preparation practices in hospitals. Improper food handling has been identified as a significant contributor to foodborne illness outbreaks in healthcare settings [40]. For instance, Ülker and Erdoğan [41] reported an outbreak in which 310 health personnel were infected following lunch prepared in a hospital kitchen, resulting in hospitalization for 13 individuals due to *Salmonella* infection.

The persistence of pathogens such as *Listeria* spp. can be influenced by various factors, including material porosity and cleaning frequencies [42]. *L. monocytogenes* can coexist and interact with multiple bacterial species found in food processing environments, complicating contamination issues. As raw foods are recognized as a source of pathogens in processing areas, the risk of listeriosis must be considered, particularly when selecting fresh produce for immunocompromised patients [42].

The findings of this study emphasize that hospital kitchens and food preparation areas are critical points for potential cross‐contamination, posing substantial risks to vulnerable patient populations [16]. For instance, Adibi et al. indicated that blenderized tube feedings in Iranian hospitals often exceeded safe bacterial limits, with pathogens such as *E. coli* and *L. monocytogenes* detected at concerning levels [17]. Another study reported an average bacterial contamination level of 5.5 × 10^6^ CFU/mL in comparable samples [43]. In contrast, research conducted by Moazen et al. assessed the microbiological quality of commercial enteral feedings in two hospitals in Shiraz, Iran, finding that microbial quality was satisfactory, with total viable counts falling below FDA standards, no detectable coliforms, and low levels of *E. coli*, suggesting that the feedings are safe for patients [44].

Gholam‐Mostafaei et al. examined the prevalence of bacterial species and their associated antimicrobial resistance profiles in isolates obtained from a hospital kitchen in Tehran, Iran. Among the 200 samples collected, *S. aureus*, *E. coli*, *Acinetobacter* spp., *Pseudomonas* spp., and *Enterococcus* spp. were identified at frequencies of 15.5%, 8%, 2.5%, 0.5%, and 0.5%, respectively [16].

Research by Greig J and Lee M indicated that 50% of deaths due to intestinal infections in healthcare centers are caused by *Salmonella* [45]. In this study, raw vegetables exhibited the highest level of contamination at 40%, followed by grilled foods at 23.3%, and mixed rice at 13.3% with *Salmonella* contamination. Plain and mixed rice, chicken, soup, and fish each had a contamination rate of 6.6%, while stewed foods showed the lowest level at 3.3%.

E. Lahou et al. examined 49 food samples prepared in a hospital for *L. monocytogenes* and microbial quality, revealing that 6 out of 49 samples (12.2%) were contaminated. These values are higher than those found in our study [46].

Ranjber et al. investigated 580 raw (red meat, chicken, fish) and cooked (red meat, chicken, fish, soup) food samples collected from hospitals in Isfahan [47]. Their results indicated that 72.6% (39 samples) exhibited contamination with *E. coli* strains, with higher contamination levels found in cooked meat and soup compared to fish and chicken. Of these contaminated samples, one cooked meat sample and one soup sample were identified as *E. coli* O157 H7 [47]. However, our findings indicated that only 4 samples (1.1% of the total) were infected with *E. coli* O157 H7, representing a lower contamination rate than reported in this study.

Additionally, Bos et al. found that the absence of hand hygiene, coupled with the preparation of large quantities of food (turkey meat), and failure to maintain appropriate temperatures from preparation to serving, resulted in the transmission of *C. perfringens* in a healthcare facility. This led to unfortunate deaths among neurological and mental patients in America. The results of these studies align with our findings regarding the contamination of hospital food samples with *C. perfringens* [48].

The study conducted by Paula Cristina et al. found no *Salmonella* contamination in any of the boiled, washed, and disinfected raw vegetable and fruit samples related to neutropenic patients. The quality of these samples met Brazilian standards. These findings contrast with our results concerning raw food samples in hospital settings [49].

Variations among studies assessing the efficacy of cleaning and sanitation procedures may stem from various factors, including the concentration of sanitizing agents, duration of contact with food, and the cleanliness of utensils used for cutting and peeling [50].

**Figure 1:**
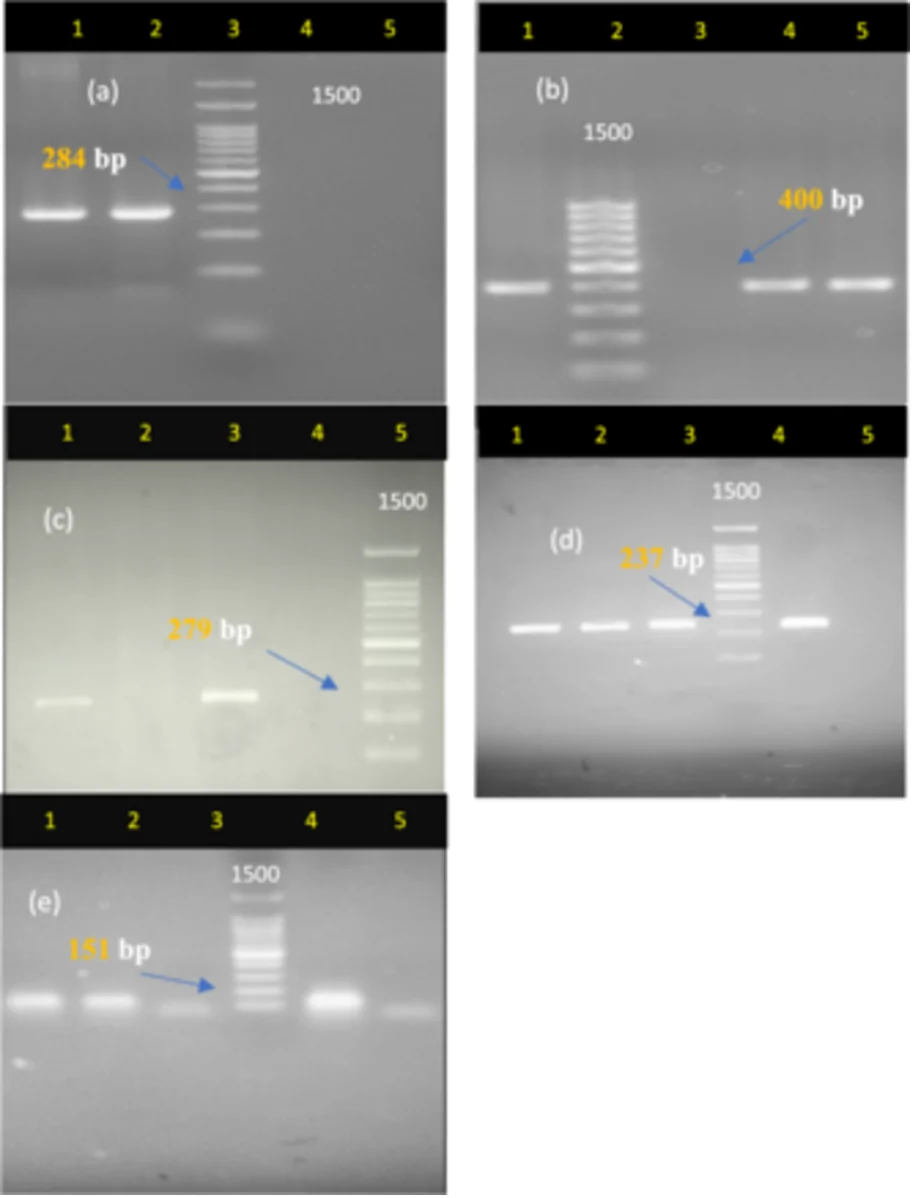
Agarose gel electrophoresis of PCR‐amplified products. Ladder: 100 bp DNA ladder (ParsTous Biotechnology, Mashhad, Iran). (a) Amplified *InvA* genes with a band length of 284 bp related to *Salmonella*. (b) Amplified *groEL* genes with a band length of 400 bp related to *B. cereus*. (c) Amplified 16S rDNA genes with a band length of 279 bp related to *C. perfringens*. (d) Amplified *prfA* genes with a band length of 237 bp related to *L. monocytogenes*. (e) Amplified *EaeA* genes with a band length of 151 bp related to *E. coli* O157 H7.

Our PCR analysis linked molecular detection to culture results (Figure 1), confirming pathogen identities. For *Salmonella*, 83 samples were culture‐positive, with 30 (8.4% of 360) confirmed via the *InvA* gene (284 bp), primarily in raw vegetables (3.3%) and koubideh (1.1%). These results indicate *Salmonella* as the dominant pathogen, likely due to its environmental resilience and association with raw produce [51]. *L. monocytogenes* had 61 culture‐positive samples, with 29 (5.8%) verified by the *prfA* gene (237 bp), mainly found in salads, grilled foods, and fish, indicating potential post‐processing contamination [52]. *E. coli* O157 H7 was detected in 4 of 20 culture‐positive samples (1.1%) via the *EaeA* gene (151 bp), predominantly in salads, consistent with fecal‐origin contamination of produce [53]. *B. cereus* was confirmed in 25 culture‐positive samples (0.5%) using the *groEL* gene (400 bp), peaking in raw vegetables and koubideh, indicating the survival of spores during food preparation [54]. *C. perfringens was* identified in 2 culture‐positive samples (0.5%) via the 16S rDNA gene (279 bp), linked to raw vegetables and fish, likely resulting from inadequate temperature control [55]. Thus, *Salmonella* was the most prevalent pathogen, while *C. perfringens* and *B. cereus* exhibited the lowest rates, associated with specific handling issues.

This finding underscores the importance of maintaining strict food safety protocols within hospital kitchens to prevent outbreaks. To mitigate contamination risks, hospitals should prioritize staff training on proper food handling techniques and enforce hygienic practices, such as regular handwashing. Additionally, ensuring that raw and cooked foods are stored at appropriate temperatures is crucial [6, 7].

To minimize the risk of pathogen transmission through raw foods such as salads and vegetables, preventing secondary contamination is essential. Secondary contamination refers to the transfer of pathogens from contaminated surfaces or foods to uncontaminated items, which can occur during food preparation, storage, or serving [56]. This process significantly increases the likelihood of infections, particularly with raw foods, as they may not undergo sufficient cooking to eliminate pathogens [56, 57]. Using aromatic herbs like dried mint as flavoring agents in prepared salads must be approached with caution, as they are minimally processed and can be significant sources of contamination, as confirmed by several studies identifying *Salmonella* [58]. Furthermore, regular microbial testing of food samples and kitchen surfaces is essential to identify potential contamination sources before food reaches patients.

### 3.1 Limitations

This study provides valuable insights into microbial contamination in hospital catering; however, several limitations must be acknowledged. The research was limited to a few teaching hospitals in Mashhad, which may not represent the microbial quality of food in all healthcare facilities in Iran. Additionally, the study solely collected samples without linking them directly to patient outcomes or clinical data, which limits the ability to assess the impact of identified pathogens on patient health. The research examined a limited selection of pathogens (*Salmonella*, *L. monocytogenes*, *E. coli* O157 H7, *B. cereus*, *C. perfringens*), potentially overlooking other relevant bacteria, such as *S. aureus*, commonly found in hospital settings, as well as broader hygiene indicators like total coliform bacteria. While PCR confirmed pathogen identity, it did not assess virulence or antibiotic resistance, both of which are critical for understanding clinical implications. Finally, the study did not evaluate specific contamination sources, including water, equipment, or personnel hygiene practices, limiting the ability to identify intervention points. Future research should address these gaps to enhance the generalizability and applicability of the findings.

## 4 Conclusion

This study revealed that 18.2% of food samples from hospital catering in Mashhad's teaching hospitals failed to meet microbial quality standards, posing a significant risk to immunocompromised patients. Raw vegetable salads exhibited the highest contamination levels, driven by *Salmonella* (8.4%) and *L. monocytogenes* (5.8%), while grilled foods ranked second, reflecting cross‐contamination risks. Molecular analysis confirmed the presence of these pathogens, linking them to culture results and highlighting raw and mishandled foods as key vectors. Given that these foods are served to a vulnerable patient population, our findings highlight an urgent public health imperative to enhance food safety practices within clinical settings. We strongly recommend the immediate implementation of a systematic Hazard Analysis and Critical Control Points (HACCP) system and mandatory, regular microbiological monitoring of hospital catering to mitigate these risks. Furthermore, based on the contamination profiles, future studies are urgently needed to assess environmental vectors, including water supply, kitchen equipment surfaces, and food handler hygiene, to precisely identify the points of biological hazard origin and ensure safe hospital catering.

## Author Contributions

**Mojtaba Raeisi:** writing – original draft, formal analysis. **Fateme Asadi Touranlou:** writing – original draft, writing – review and editing. **Golshan Shakeri:** writing – review and editing. **Maliheh Doustinouri:** investigation, methodology, conceptualization, data curation. **Arefeh Erfani:** investigation, writing – review and editing. **Asma Afshari:** writing – review and editing, methodology. **Mohammad Hashemi:** supervision, project administration, writing – review and editing. All authors have read and approved the final version of the manuscript. Mohammad Hashemi had full access to all of the data in this study and takes complete responsibility for the integrity of the data and the accuracy of the data analysis.

## Funding

The authors have nothing to report.

## Conflicts of Interest

The authors declare no conflicts of interest.

## Transparency Statement

Mohammad Hashemi affirms that this manuscript is an honest, accurate, and transparent account of the study being reported; that no important aspects of the study have been omitted; and that any discrepancies from the study as planned (and, if relevant, registered) have been explained.

## Data Availability

The data that support the findings of this study are available from the corresponding author upon reasonable request.
